# Stress in wildlife: comparison of the stress response among domestic, captive, and free-ranging animals

**DOI:** 10.3389/fvets.2023.1167016

**Published:** 2023-04-17

**Authors:** Mina Cansu Karaer, Nina Čebulj-Kadunc, Tomaž Snoj

**Affiliations:** ^1^Food and Agriculture Vocational School, Çankiri Karatekin University, Çankiri, Türkiye; ^2^Veterinary Faculty, Institute of Preclinical Sciences, University of Ljubljana, Gerbičeva, Ljubljana, Slovenia

**Keywords:** stress response, wildlife, captive animals, domestic animals, glucocorticoids

## Abstract

The stress response, which involves joint activity of the nervous and endocrine systems, is one of the basic adaptive mechanisms that ensures the survival of the individual. The activation of the sympathetic nervous system, the sympathetic-adrenal-medullary axis, and the hypothalamic–pituitary–adrenal axis enables organisms to respond to endogenous and exogenous challenges. Repeated short-term stress leads to long-term stress, which disrupts physiological homeostasis. Unlike domestic animals, wild animals are not protected from environmental and weather influences or treated for diseases. In addition, climate change, habitat fragmentation and loss, and urban stressors (such as light, noise and chemical pollution; xenobiotics; traffic; and buildings) affect individual wildlife and populations. In this review, we have attempted to depict the magnitude of the stress response in wildlife and related domestic animals as well as in captive and free-ranging animals. The intensity of the stress response can be estimated by determining the concentration of glucocorticoids in body fluids, tissues, and excreta. A comparison of results from different studies suggests that domestic animals have lower fecal and hair glucocorticoid concentrations than related wild animals. Additionally, fecal and hair glucocorticoid concentrations in captive animals are higher than in free-ranging animals of the same species. As there are limited data on this topic, we cannot draw definitive conclusions about glucocorticoid concentration and stress response. Further studies are needed to clarify these issues.

## Introduction

The concept of the fight-or-flight response and the notion of homeostasis were introduced by Harvard physiologist Walter Bradford Cannon in 1929 (1). Since then, the pathophysiological effects of stress have been extensively studied. Selye (2) described stress as a nonspecific response of the organism to a harmful factor. Selye (2) also introduced the general adaptation syndrome and divided the overall response to stress into three phases: alarm, resistance, and exhaustion. When individuals are exposed to a stressor, the alarm reaction is triggered, followed by the stage of resistance to maintain homeostasis; finally, in the stage of exhaustion, individuals succumb to the stressor. Today, stress is defined as the effect of all abiotic and biotic factors that can negatively affect the performance of an individual and alter the psychological, physiological and/or physical health of living organisms (3–5).

The current stress framework is broader than the classic approach. All organisms, including unicellular organisms and plants, have systems to protect against harmful factors; the purpose of these systems is to maintain homeostasis (6). Protective systems are activated when an endogenous or exogenous stimulus disrupts what Claude Bernard called the “milieu interieur” of an organism. At the cellular level, some highly potent metabolites called reactive oxygen species (ROS) are formed. ROS play a role in cell signaling but can also react with various organic cellular molecules to cause oxidative stress, which can lead to lipid and protein peroxidation and severe damage to bioactive molecules (7–9). Cells have an antioxidant capacity to protect against the effects of ROS. In fact, the mechanisms protecting against oxidative stress are not directly related to the stress response in higher organisms. However, oxidative stress can also be considered a stressor since it disrupts cell homeostasis. Thus, the stress effect is generally considered any disruption of homeostasis that may occur at the cellular or tissue level as well as a complex response of all organ systems.

As described by Lu et al. (10), the stress system consists of a stressful stimulus, stressor, stress, stress response, and stress effect. Therefore, the framework of stress includes exogenous factors and endogenous responses that eventually lead to the stress effect. The stressor → stress → stress response cascade ends in the stress effect. A stressor is a stimulus that threatens homeostasis and triggers a response to restore homeostasis (11, 12). The stress response, which emerges as a result of the joint activity of the nervous and endocrine systems, is one of the basic adaptive mechanisms to ensure the survival of the individual. From an evolutionary perspective, the stress response has evolved as a protective mechanism necessary for the survival of the species, thus ensuring its evolutionary conservation. The most frequently mentioned response to stressful situations in the animal world is the fight-or-flight response, to which the freeze response can also be added (13). Thus, in stressful situations, both predators and prey respond with intense physical activity triggered by stimulation of the sympathetic-adrenal-medullary (SAM) axis and the hypothalamic–pituitary–adrenal (HPA) axis. In addition to the predator-prey relationship, stress can also be triggered by various other exogenous and endogenous stimuli, such as weather conditions, food deprivation, diseases, ectoparasite infestations, reproductive activity, parturition, and social relationships. Considering the abovementioned stressful stimuli, it is obvious that the stress response can be divided into non-threatening (“good”) stress or eustress and harmful (“bad”) stress, called distress. Therefore, the general belief that all forms of stress have a negative impact on homeostasis is not correct (12). Living organisms respond to internal or external stressors to which they are constantly exposed. The body, on the other hand, analyzes and responds to the events that cause stress. Since eustress does not affect animal health, it is studied (less frequently) at the physiological level, while distress is studied in depth as a pathophysiological process that causes severe problems in intensive livestock production and in humans.

Wild animals are frequently or constantly exposed to stressful stimuli, as they are all either predator or prey. Unlike domestic animals, wild animals are exposed to environmental stress, food deprivation, parasite infestation and numerous untreated diseases. In addition, human pressure, wildlife management interventions, habitat fragmentation and loss, climate change, poaching, and invasive species are also stressors to wildlife populations (4, 14, 15). Therefore, stress in wildlife can be considered different from than in related domestic species. Furthermore, stress affects the growth, development, and survival of organisms and ultimately influences wildlife population dynamics (5).

In this review, we attempted to describe the stress response and compensatory mechanisms for adaptation to short-term stress and the effects of long-term stress. In addition, we tried to present the magnitude of the stress response in wildlife and related domestic species and free-ranging and captive animals.

## Stress response

Stressors evoke a series of reactions in organisms. Sensory organs or cells detect information about the stressful stimulus, which is transmitted via sensory nerves to cortical centers in the brain. The information is further transmitted through the limbic system and hypothalamus, thus stimulating the sympathetic nervous system and activating the HPA axis (16).

### Response of the sympathetic nervous system

The first response following the perception of a stressor is activation of the sympathetic nervous system, which leads to two different but related pathways, i.e., sympathetic neurotransmission and activation of the SAM axis. The first pathway, which is faster, is neurotransmission in efferent peripheral sympathetic adrenergic nerves, leading to the release of noradrenaline at the neuroeffector junction. There, as a neurotransmitter, noradrenaline binds to the postsynaptic membrane α and β adrenergic receptors (with a higher affinity for α. α receptors (subtypes α1, α2, and α3) are found in cells of almost all organ systems. In vascular smooth muscle cells, they control baseline vascular tone and modulate systemic vascular resistance and venous capacitance. In the gastrointestinal tract, activated receptors provide vasoconstriction and slow digestive processes (17, 18). In addition to the effects mentioned above, activation of α1-receptors influences cognition, which is also an important factor in the stress response (19). Furthermore, neurotransmission in cholinergic preganglionic sympathetic nerves innervating the adrenal medulla and derived from splanchnic nerves activates the SAM axis (20). In the neuroeffector junction of medullary chromaffin cells, acetylcholine binds as a neurotransmitter to cholinergic receptors on the postsynaptic membrane and provides adrenalin secretion (21). Endogenous ligands for β adrenergic receptors (subtypes β1, β2, and β3) are catecholamines with adrenaline as the ligand with the highest affinity. Therefore, the receptors are activated after the release of adrenaline from the adrenal medulla. Adrenaline is released into the systemic circulation as a hormone and reaches target cells through the blood. Hence, the effects of the SAM axis occur a few moments later than the direct effects of the sympathetic nervous system. Increased heart rate, increased blood pressure, bronchodilatation, intense blood flow to the lungs and skeletal muscles, glycolysis, and gluconeogenesis during the fight-or-flight response are the result of the activated sympathetic nervous system and are maintained by the SAM axis.

### Activation of the HPA axis

In the next step of the stress response, the HPA axis is activated. The HPA axis is highly important to homeostasis as it responds to environmental variables that cause stress. It is a complex system of neuroendocrine pathways and regulatory circuits that are activated under stressful conditions (22). Glucocorticoids are steroid hormones that are released according to circadian rhythms as well as during stress. They influence cellular functions and are involved in both anabolic and catabolic reactions (23). In mammals, with the exception of rodents where corticosterone is present in high concentration, cortisol is the major glucocorticoid hormone affecting many different systemic activities and is secreted by the adrenal glands (24, 25). Activation of the HPA axis begins with the secretion of corticotropin-releasing hormone (CRH) from the paraventricular nucleus of the hypothalamus. CRH triggers pituitary stimulation and subsequent release of adrenocorticotropic hormone (ACTH) into circulation. ACTH binds to a G-protein-coupled receptor, the melanocortin-2 receptor. This binding leads to activation of adenyl cyclase, production of cAMP, and activation of protein kinase A (PKA). PKA alters the activity of certain transcription factors by phosphorylation. ACTH activates HMG-CoA reductase, increases LDL-C ester uptake, and activates hormone-sensitive lipase and acyl-coenzyme A (CoA) to increase the cholesterol pool for steroidogenesis. The primary site of ACTH action is the adrenal cortex, which produces glucocorticoids, mineralocorticoids, and androgens. When the adrenal cortex is stimulated by ACTH, the conversion of cholesterol to steroids occurs in a series of enzymatic steps, and cortisol is synthesized from pregnenolone through a series of isomerization and hydroxylation steps catalyzed by 11β-hydroxylase (26, 27). Cortisol is released into the blood and binds to corticosteroid-binding globulin (23, 28). The increase in cortisol levels in the body is sensed by the hypothalamus and pituitary receptors and regulated by negative feedback.

Cortisol exerts effects in organisms upon binding to intracellular glucocorticoid receptors (GRs). In cell signaling, the glucocorticoid-GR complex activates glucocorticoid-responsive elements in promoter or enhancer regions in a DNA sequence-specific manner to cause transcriptional induction of target genes or binds to negative glucocorticoid-responsive elements (29). The glucocorticoid-GR complex plays an important role in gene expression of kinase enzymes involved in the catabolism of carbohydrates, proteins, and lipids, but cortisol also has anabolic effects. Glycogenolysis during hepatic glucose production and gluconeogenesis (the anti-insulin effects of cortisol) are the result of the transcription of enzymes involved in these metabolic pathways. In addition, inhibition of the transcription of proinflammatory cytokines and an altered acquired immune response are the result of cortisol activity (13).

Thus, the activity of both the sympathetic nervous system and the HPA axis influence the responses of organisms that lead to adaptation to stress.

### Short- and long-term stress

The fight-or-flight response (also known as the acute stress response) is a physiological response that occurs due to a perceived harmful event, attack, or life-threatening danger (stressor) (1). The fight-or-flight response is based on the theory that the sympathetic nervous system of organisms responds to stressors and prepares for fight or flight. In doing so, it elicits a series of responses through the actions of noradrenaline and adrenaline, two transmitters that act through α- and β-adrenergic receptors. These responses can occur in a variety of organ systems. The stress response includes increases in heart rate, increases in blood pressure and respiratory rate, and decreases in gastrointestinal tract activity (13, 21). These integrated physiological changes are a part of the fight-or-flight response observed following acute stressors.

Short-term glucocorticoid effects in the face of stressful situations (temporary climatic events, predator threats, dominance interactions) are referred to as acute stress and are adaptive, but when the effects of stressors extend over a longer period of time (resource limitation, famine, drought), chronic stress ensues, with more severe consequences (3, 4, 30).

When the stressful stimulus lasts for a long time, the HPA axis remains active. In such a situation, negative effects of glucocorticoids are detected (16). For example, long-term or chronic stress can lead to a negative feedback loop in the HPA axis, resulting in a downregulated response to any further acute stressor (31). Longitudinal studies and regular examinations are necessary to detect chronic stress. Such approaches, evaluating the population and stressors over the long term, are more common in the fields of wildlife management and planning (30, 32).

## Estimation of stress according to glucocorticoid concentrations (a stress biomarker)

Animal species respond differently to different types of glucocorticoids. For example, the responses of certain animals to cortisol (the primary glucocorticoid in fish and most mammals) (3, 33) and corticosterone (the primary glucocorticoid in rodents, birds, reptiles, and amphibians) (34) can vary depending on age, sex, developmental stage, and body condition. In this review, we focused mainly on mammalian species to provide data on cortisol as a stress biomarker. The magnitude and patterns of elevated cortisol levels vary not only among species and sexes, but also among individuals. The differences in responses may be caused by genetic differences (35).

Determination of blood cortisol (or corticosterone) concentrations can be used to assess stress responses in animals. The blood cortisol concentration indicates the current glucocorticoid status. As shown in the study by Caroprese et al. (36), cortisol concentration increases rapidly after a stressful stimulus and can be fourfold higher than baseline in a couple of minutes after the stressful stimulus. Because blood cortisol concentrations vary daily and concentrations vary among individuals, it is difficult to determine the stress response based on a single blood cortisol measurement. In addition, blood collection and manipulations of the animal cause stress and may mask the true glucocorticoid status. Therefore, the determination of blood glucocorticoid levels as a stress biomarker is not recommended as an appropriate tool for assessing the stress response. Non-invasive methods of sampling are preferable because they prevent both the negative impact on animal welfare and the stress caused by capturing/restraint of the animals, which can be misleading when evaluating samples (37, 38).

Glucocorticoids are mostly metabolized and inactivated in the liver and excreted from the body through bile to the feces. They may also be found in urine, milk, saliva, and hair. In these materials, they are present in free and metabolized forms (39, 40). Due to the different dynamics of cortisol excretion in different materials, choosing the right material for analysis is important to gain insight into the cortisol status. Salivary cortisol levels indicate the current glucocorticoid status and are subject to circadian fluctuations similar to blood cortisol levels. Concentrations in saliva are approximately 10-fold lower than that in plasma (41). As samples can be collected non-invasively, salivary cortisol concentrations can be used to assess short-term stress (41). Evaluation of fecal glucocorticoid concentrations is used to assess stress in many animal species (42–45). Fecal glucocorticoid concentrations reflect the glucocorticoid status of the animal over one to 2 days or more, depending on the gut transit time, prior to sampling. The method is useful for assessing short- and long-term stress. More reliable results are obtained if the samples are taken several times at intervals of several days. Fecal samples offer several non-negligible advantages: they can be collected easily and without irritating the animal (46). Thus, this method provides the opportunity to take samples frequently, even over long periods of time.

Moreover, during hair growth, the hair shaft is loaded with cortisol. As hair growth is a long-lasting process, the determination of the hair glucocorticoid concentration can be used to evaluate long-term stress as well as to assess animal welfare (47–49). As suggested by Sheriff et al. (50), sometimes more than one material can be analyzed to obtain a correct result. Although the detection of glucocorticoids in feces or hair provides indirect insight into glucocorticoid status, the results should be considered a retrospective aspect of the stress response.

To ensure the reliability of results, methods for detecting glucocorticoids in feces and hair must be validated for the species of interest because the choice of substrate and hormone metabolite is important for accurate detection of biological changes in stress hormone levels.

## Environment and wildlife

Climate change, habitat fragmentation and loss affect wildlife populations just as much as they affect the environment (51). For this reason, these three factors dominate the One Health Paradigm (4, 52). One Health's approach is to tackle health problems at the human-animal-environment interface, including zoonotic diseases; to develop new concepts about pathogens, comparative immunology, and epidemiological dynamics; and to understand the role played by environmental factors in each of system (53, 54). This approach illustrates the intertwined nature of diseases among humans, livestock, and wildlife. Moreover, these relationship are affected by some factors, such as host, pathogen, environmental, political, and socioeconomic factors (53). As the human population is increasing worldwide, animal populations are also growing significantly. Therefore, human–wild animal conflict and interaction are ineluctably on the rise. It is very important to prioritize the One Health perspective in studies of wild animals. In addition, the increase in the human population is significantly changing the environmental conditions on Earth, which may expose some species to urban stressors such as light pollution, noise pollution, chemical pollution, traffic, buildings, and fragmented habitats, as well as other factors that may be beneficial, such as anthropogenic food supplies and warmer microclimates, especially during the colder months (55–57). These environmental stressors, which we call urban stressors, can be foreseeable or unforeseeable and greatly impact individual fitness and evolutionary adaptation. Especially because urban landscapes are managed primarily for humans, these factors can pose significant challenges to wildlife species. The stressful environments may cause morphological, physiological and parasitological differences between urban and non-urban populations (58, 59).

The HPA axis is the primary mechanism that boosts the adaptive response to environmental stressors in vertebrates (Table 1) (71). The physiological coping mechanism used by vertebrates to ensure survival under unfavorable environmental conditions is the acute stress response, which is characterized by the release of glucocorticoid hormones. The short-term secretion of the aforementioned steroid hormones can be beneficial; however, secretion of the same hormones over long periods has side effects on reproductive activity, the immune system and brain functions. Therefore, many species that inhabit urban ecosystems or urban areas cannot survive unless they their stress response adapts to the conditions of the city (56). However, while some studies have supported this idea and showed altered animal behavior and increased glucocorticoid concentrations, other studies have shown that, in contrast, stress responses decrease with human activity. Thus, studies have reported that although some wildlife species are negatively affected by anthropogenic disturbances (72, 73) and their populations decrease, some species exhibit no effects (74), and some exhibit a decreased physiological stress response (73) and may even benefit from urban conditions (56, 75). These differences among studies may be related to the complex responses of the neuroendocrine system to chronic stress, which may limit the physiological capacity to respond to the stressors, as well as to differences in species, populations, individuals (in terms of physiology and sex), and urban environments (50, 76, 77). Differences between populations are referred to as “synurbization”, i.e. adaptation to urban ecosystems (58, 59). However, the role of potential regulatory factors has not been formally and thoroughly studied.

**Table 1:** Fecal glucocorticoid concentrations in wild animals and related domestic animal species.

**Animal species**	**Fecal glucocorticoids (ng/g)**	**References**
Wild boar *(Sus scrofa)*	142–684	(60)
Domestic pig *(Sus scrofa)*	24.36 ± 2.02 17.32 ± 1.20	(61)
Wild bison *(Bison bison)*	100–600^*^	(62)
Domestic bulls *(Bos indicus)*	8.6–116	(63)
Domestic cows *(Bos taurus)*	10.14–54.83	(64)
Gray wolf *(Canis lupus)*	116–1270	(65)
Dog *(Canis canis)*	190–601	(66)
Dog *(Canis canis)*	1.16 ± 0.23	(67)
European wildcat *(Felis silvestris)*	2–5^*^	(68)
Cat *(Felis catus)*	0.83 ± 0.08	(67)
Wild rat *(Rattus norvegicus)*	38.1–98.2	(69)
Laboratory rat *(Rattus norvegicus)*	5–7^*^	(70)

^*^Results were obtained from figures and indicate approximate concentrations.

Beyond the direct effects on wildlife, urbanization may have more devastating effects on wild populations in the future. As a result of urbanization, epidemiological processes may increase the morbidity of infectious diseases, which may impose substantial barriers to the conservation of wildlife populations and human health.

## Fecal and hair glucocorticoids concentrations in free-ranging and captive animals and their related domestic animal species

The designs of the studies included in this review are very different. The methods of sampling, glucocorticoid extraction from samples, and glucocorticoid detection vary from study to study. In most studies, cortisol or corticosterone concentrations were measured before and after stress exposure or ACTH challenge. The age and reproductive status of the animals also varied among studies, and several studies did not include both sexes. In addition, studies were conducted under different climatic conditions, and the timing of sampling varied among studies, which may influence the interpretation of the results, since daily and seasonal fluctuations of glucocorticoids have been observed (78–80). The studies also varied in terms of analytes since cortisol or glucocorticoid metabolites were determined in fecal samples. Additionally, in some studies, hair was obtained from dead animals. Therefore, the studies cannot be directly compared. When data are available, we report the range of cortisol, corticosterone or glucocorticoid metabolites concentrations; otherwise, mean values are reported.

### Comparison of glucocorticoid concentrations in wild and related domestic species

Comparing the results of studies examining glucocorticoids in the feces or hair of domestic and wild species, glucocorticoid concentrations are higher in wild animals than in related domestic species. Tables 1, 2 display data from studies showing concentrations of glucocorticoids in the feces or hair of various domestic and wild animal species. Although a conclusion cannot be drawn due to differences in analytical approaches, the data are indicative of higher glucocorticoid levels in wild animals compared to domestic ones.

**Table 2:** Hair glucocorticoid concentrations in wild animals and related domestic animal species.

**Animal species**	**Hair glucocorticoid concentration (ng/g)**	**References**
Wild boar *(Sus scrofa)*	4.2–51.4	(81)
Domestic pig *(Sus scrofa)*	0.49–8.92	(82)
Muskox *(Ovibos moschatus)*	20–90^*^	(83)
Domestic cattle *(Bos taurus)*	8.1–15.9	(84)
Gray wolf *(Canis lupus)*	1.6–108.8	(85)
Dog *(Canis canis)*	13.3–20.2	(66)
Dog *(Canis canis)*	2.10 ± 0.22	(67)
European wildcat *(Felis silvestris)*	3.57–8.91	(86)
Cat *(Felis catus)*	0.2–251.5	(87)
Cat *(Felis catus)*	3.32 ± 0.27	(67)

^*^Results were obtained from figures and indicate approximate concentrations.

Wild boars and domestic pigs belong to the same species *(Sus scrofa);* thus, their metabolic status can be easily compared. The data from free-ranging wild boars (Table 1) were obtained from different tiger reserves in India (60). As is evident in Table 1, their fecal levels were higher than those obtained from domestic pigs. In addition, hair glucocorticoid concentrations (shown in Table 2) were also higher in wild boars. These samples were obtained from wild boars in Japan captured 10 years after the nuclear accident near the power plant in Fukushima, Japan (81). As was reported in the study, glucocorticoid levels in the hair were not affected by the increased radiation, but the glucocorticoid levels were higher than in domestic pigs (82).

Wild bison and domestic cattle belong to different but related species (*Bison bison* and *Bos taurus*, respectively). We compared fecal glucocorticoid levels in these species because data on wild *Bos taurus* are not available. The range of glucocorticoid concentrations in bison bulls correlates with their hierarchy rank; bulls with high social status must pay the physiological price of high glucocorticoid levels (62). Furthermore, Tallo-Parra et al. (64) measured fecal concentrations in Holstein cows (*Bos taurus*), while Hernandez-Cruz et al. (63) measured fecal glucocorticoid concentrations in zebu bulls (*Bos indicus)* of different ages from beef farms. Because of differences in species, design, and sex (males have higher cortisol levels), the studies cannot be directly compared. However, the higher fecal glucocorticoid concentrations in the wild bison population (Table 1) may support the hypothesis of a more active HPA axis in wild animals. Additionally, comparison of hair (or qiviut) glucocorticoid concentrations in two related species, domestic cattle and wild muskoxen (*Ovibos moschatus*), also revealed higher levels in the wild population (64, 83, 84).

In addition, we compared the glucocorticoid levels in some carnivores. The gray wolf and the dog are phylogenetically related, as both belong to the same genus (*Canis*), but the species are different (*Canis lupus* and *Canis canis*). In free-ranging wolves, fluctuations in fecal glucocorticoid levels have been observed. Several exogenous and endogenous factors, such as age, sex, reproductive activity, and season, influence the intensity of glucocorticoid secretion. Fecal glucocorticoid concentrations also greatly increased in the presence of humans (65). In dogs from animal resource centers, fecal glucocorticoid levels appear to be similar, but unlike wolves, high elevations in response to humans were not observed (66). Similarly, comparable hair glucocorticoid concentrations were found in many animals of both species, but only wolves were found to have very high levels. Moreover, compared to the findings of Bryan et al. (66), Accorsi et al. (67) reported lower physiological glucocorticoid levels in the feces and hair of dogs.

In the European wildcat (*Felis silvestris*), no significant differences in fecal glucocorticoid levels were found between populations from different habitats (68). However, fecal glucocorticoid levels were higher than those in domestic cats (67). As described by Franchini et al. (86), hair glucocorticoid concentrations are higher in European wildcats than in feral cats. Interestingly, Contreras et al. (87) reported that hair glucocorticoid levels depend on the body region where the sample was obtained. The lowest levels of glucocorticoids were found in hair samples from the neck. Hair can be contaminated with saliva and salivary cortisol; thus, significant differences were found in hair samples from different body regions. This explains the wide range of hair glucocorticoids concentrations in Table 2.

Even laboratory rats, which are somewhat domesticated, have lower fecal glucocorticoid levels than their wild counterparts (69, 70).

While we cannot draw a firm conclusion due to the differences in research design and study objectives, the data show higher cortisol levels in wild species. As mentioned earlier, wild individuals are either predator or prey and are exposed to various environmental and social stressors. Although the data in Tables 1, 2 originated from studies with different approaches, we hypothesize that wild animals are exposed to more stressful situations, which is reflected in higher glucocorticoid levels.

### Comparison of glucocorticoid concentrations in free-ranging and captive animals

Data about stress and glucocorticoid concentrations in free-ranging animal species that also live in captivity are limited. Comparison is possible only in the species we mention in Tables 3–**5**.

**Table 3:** Fecal glucocorticoid concentrations in some captive and free-ranging species.

**Animal species**		**Fecal glucocorticoid concentrations (ng/g)**	**Reference**
Spotted hyena	Captive	4.6–377.4	(88)
(*Crocuta crocuta*)	Free-ranging	35.5–112.6	(89)
Canada lynx	Captive	80–450^*^	(90)
(*Lynx canadensis*)	Free-ranging	40–200^*^	(90)
Cheetah	Captive	196.08 ± 36.20	(91)
(*Acinonyx jubatus*)	Free-ranging	71.40 ± 14.35	(91)
Fallow deer	Captive	95.0–3271	(92)
(*Dama dama*)	Free-ranging	10.0–2035	(79)
Asian elephant (*Elephas maximus*)	Captive Free ranging	200–6000^*^ 100–2500^*^	(93, 94)
Lemur	Captive	50.0–125.0	(95)
(*Lemur catta*)	Free-ranging	16.98 ± 1.10–46.77 ± 1.15 ^**^	(96)
Yucatan spider monkey	Captive	1925.4 ± 252.2	(97)
*(Ateles geoffroyi yucatanensis)*	Free-ranging	1224.2 ± 168.5	(97)
Gilbert's potoroo	Captive	7^*^	(98)
*Potorous gilbertii*	Free-ranging	12^*^	(98)

^*^Results were obtained from figures and indicate approximate concentrations. ^**^In the study by Pride (96), the results were in logarithm scale. This table presents the antilogarithm values.

As shown in the study by Benhaiem et al. (88), the range of fecal glucocorticoid levels in juvenile spotted hyenas (*Crocuta crocuta*) is broader, and levels are higher than in free-ranging females (89). However, a comparison of the mean values of the two studies revealed a different situation. In captive juveniles of different ages, fecal glucocorticoids were found at concentrations of 18.9 and 51 ng/g, whereas the concentrations in nonlactating and lactating free-ranging females were 40 and 112 ng/g, respectively. Young, pregnant, and lactating animals are known to have high glucocorticoid levels, so lower levels would be expected in other animal categories. Unfortunately, data are not available for other categories. Because of this variation, the studies cannot be directly compared; although mean glucocorticoid levels were higher in free-ranging animals, this cannot be considered a conclusion. Two Felinae species, the Canada lynx (*Lynx canadensis*) and cheetah (*Acinonyx jubatus*), showed similar fecal glucocorticoid patterns. In both species, captive animals had higher fecal corticosteroid levels than free-ranging animals (90, 91). In captive cheetahs, morphological evaluation of the adrenal glands showed evidence of increased activity suggestive of chronic stress (91). There are many factors that influence fecal corticosteroid concentration, thus we cannot definitively conclude that animals in captivity are more stressed than those in the wild. To confirm that the higher glucocorticoid levels in captive animals are a result of a stress response, further studies should be conducted to look at the activity of the SAM axis in addition to glucocorticoid concentration and HPA axis activity. It is important to note that while the lifespan of some captive species is longer due to preventive health care, adequate nutrition, and controlled environments, alterations in behavior may be indicative of a questionable welfare state (113).

Studies of glucocorticoid status in free-ranging and captive fallow deer *(Dama dama)* used the same method of fecal glucocorticoid determination and were also similar in design (79, 92). In both studies, glucocorticoid status was followed over a one-year period. As shown in Table 3, captive deer had a wider range of glucocorticoid levels. However, median levels were also several times higher in captive deer (79, 92).

Similarly, the Asian elephant has also been found to have higher fecal glucocorticoid concentrations in captivity (93, 94).

In addition, two primate species, lemur and Yucatan spider monkey, have been found to have higher fecal glucocorticoid concentrations in captive animals (95–97), although the studies by Pollastri et al. (95) and Pride (96) reporting glucocorticoid concentrations in lemurs are not directly comparable.

In contrast, in the Australian endangered marsupial, Gilbert's potoroo, higher glucocorticoid level was observed in free-ranging animals (98). Therefore, even though many studies show that captive populations of wild animals generally have higher glucocorticoid concentrations than their free-ranging counterparts, it is not definitive information unless comparable data are obtained. For instance, although not directly relevant to our research topic, according to a study by Shimamoto et al. (114), there are no significant differences in fecal glucocorticoid concentrations in Eurasian red squirrels' (*Sciurus vulgaris*) populations in rural and urban areas.

Considering the results of the studies shown in Table 3, we concluded that observed captive animals had higher fecal glucocorticoid levels than their wild counterparts.

Fecal glucocorticoid concentration, an indicator of cortisol status, reflects total glucocorticoid secretion, which can be used to assess the intensity of stressful events over a period of 1 to 2 days, whereas hair glucocorticoid concentration reflects long-term cortisol status. As shown in Table 4, similar to fecal glucocorticoid concentrations, hair glucocorticoid concentrations are also higher in captive than in free-ranging animals. Although the literature contains much information on hair glucocorticoid concentrations in animals, there are few studies directly comparing captive and free-ranging populations, which makes it challenging to draw a conclusion.

**Table 4:** Hair glucocorticoid concentration in some captive and free-ranging species.

**Animal species**		**Hair glucocorticoid concentrations (ng/g)**	**References**
Chimpanzee	Captive	0.8–15.8	(99)
(*Pan troglodytes*)	Free-ranging	0.035–5.43	(100)
Polar bear	Captive	2.31–24.0	(101)
(*Ursus maritimus*)	Free-ranging	0.16–2.26	(102)
Reindeer	Captive	1.0–5.0	(103)
(*Rangifer tarandus spp*)	Free-ranging	0.85–3.67	(104)
Common marmoset	Captive	2,710–8,906	(105)
(*Callithrix jacchus*)	Free-ranging	246.51–4,295	(106)

In contrast, blood cortisol concentrations reflect the current cortisol status. As shown in Table 5, free-ranging animals had higher cortisol levels than captive animals. These data appear to display the opposite pattern of fecal glucocorticoid levels; however, a definitive conclusion cannot be reached since the data are scarce. These results also suggest that captive animals are used to the presence of humans; thus, during blood collection, they do not respond with an intense stress response. In contrast, blood sampling of wild animals is a drastic procedure that requires manipulation of the animal and can elicit a strong stress response (37, 38). Perhaps this explains why few data are available. However, considering the higher fecal glucocorticoid levels of captive animals, we assume that the sum of all stress responses during the day that lead to activation of the HPA axis and excretion of cortisol (glucocorticoid metabolites) is higher in captive animals than in free-ranging individuals. We are aware that the comparison of some studies is not entirely reliable because the sensitivity of the methods used differs between studies, for example, in the study by Seal and Mech (107) and Thoresen et al. (108), which we consider a shortcoming of this review. Unfortunately, we were also unable to compare fecal glucocorticoids with hair or plasma glucocorticoid concentrations in the same species because of the lack of data. Therefore, comparison of the stress response between free-ranging and captive animals is a topic for further research.

**Table 5:** Plasma cortisol concentration in some captive and free-ranging species.

**Animal species**		**Plasma cortisol concentrations (ng/mL)**	**References**
Gray wolf	Captive	2.0–58.0	(107)
(*Canis lupus*)	Free-ranging	23.92–129.41	(108)
Bottlenose dolphin	Captive	2.58–14.03	(109)
(*Tursiops truncatus*)	Free-ranging	1.0–74.0	(110)
Giraffe	Captive	357,000	(111)
(*Giraffa camelopardalis*)	Free-ranging	686,000	(111)
Common degu	Captive	70–240^*^	(112)
(*Octodon degus*)	Free-ranging	10–150^*^	(112)

^*^Results were obtained from figures and indicate approximate concentrations.

An interesting question that currently lacks a no precise answer is why the natural lifespan of domestic animals is longer than that of related wildlife species and whether this is related to the stress response. If we consider glucocorticoid levels in wild animals to be species specific, domestication influenced the stress response. We believe that the HPA axis may also respond with the same intensity in domestic animals as in related wild animals, but the benefits of domestication and animal care provided by humans protect domestic animals from intense stressors. The question is what parameter(s) of domestic animal welfare facilitate longer lifespans. It has been reported that free-ranging animals with faster pace of life are subject to several environmentally-driven mortality and therefore live longer in captivity (zoos). In contrast, animals with slower pace of life live longer in natural environments (113). Thus, this review raises the question of whether frequent or chronic stress (among other external and internal factors) contributes to shorter lifespans in free-ranging species with faster pace of life.

In general, long-term stress or frequent exposure to stressors leads to activation of the SAM and HPA axes. In the long term, this can affect cardiovascular, digestive, immune as well as other systems (13, 17–19). Frequent stress in wildlife may be one of the reasons for their shorter lifespan compared to that of their domestic relatives. However, this hypothesis has not yet been confirmed and needs further investigation.

On the other hand, captive animals have higher glucocorticoid levels and longer lifespans than free-ranging animals. This information suggests benefits of human care, although the wellbeing of captive animals remains questionable.

## Conclusion

In conclusion, a review of the available literature on glucocorticoid concentration shows higher levels in wild compared to related domestic animals. In addition, captive animals have higher glucocorticoid levels than their wild counterparts. While these are interesting trends, further studies are needed to investigate whether higher cortisol levels in wildlife compared to domestic animals and in captive compared to free-ranging animals are due to a stronger or more prolonged stress response.

## Author contributions

MCK and TS: conceptualization, investigation, editing, and writing. NČK: editing and final validation. All authors contributed to the article and approved the submitted version.
